# Photodynamic Therapy for Colorectal Cancer: An Update and a Look to the Future

**DOI:** 10.3390/ijms241512204

**Published:** 2023-07-30

**Authors:** José A. Rodrigues, José H. Correia

**Affiliations:** 1 CMEMS-UMinho, University of Minho, 4800-058 Guimarães, Portugal; higino.correia@dei.uminho.pt; 2 LABBELS—Associate Laboratory, 4800-122 Braga, Portugal

**Keywords:** photodynamic therapy, colorectal cancer, photosensitizers, light sources, combination therapies, nanotechnology

## Abstract

This review provides an update on the current state of photodynamic therapy (PDT) for colorectal cancer (CRC) and explores potential future directions in this field. PDT has emerged as a promising minimally invasive treatment modality that utilizes photosensitizers and specific light wavelengths to induce cell death in targeted tumor tissues. In recent years, significant progress has been made in understanding the underlying mechanisms, optimizing treatment protocols, and improving the efficacy of PDT for CRC. This article highlights key advancements in PDT techniques, including novel photosensitizers, light sources, and delivery methods. Furthermore, it discusses ongoing research efforts and potential future directions, such as combination therapies and nanotechnology-based approaches. By elucidating the current landscape and providing insights into future directions, this review aims to guide researchers and clinicians in harnessing the full potential of PDT for the effective management of CRC.

## 1. Introduction

Colorectal cancer (CRC) is the third most common cancer worldwide (second most common cancer in women and third in men) and the second most common cause of cancer death. The widespread use of colonoscopy has led to an increase in the diagnosis of CRC in both early and late stages and a decrease in the mortality rate [1,2,3]. According to the World Health Organization (WHO), in 2020, there were 1.93 million newly diagnosed cases of CRC worldwide, resulting in 916.000 deaths attributed to CRC [4]. Generally, in CRC, 71% are in the colon and 29% in the rectum. [1]. Several factors contribute to the development of CRC. Age (over 50 years old), family history of CRC, and certain inherited genetic conditions, such as Lynch syndrome and familial adenomatous polyposis (FAP), increase the risk. Lifestyle factors, including a low-fiber and high-fat diet, consumption of red meat, consumption of processed meat, sedentary lifestyle, obesity, and smoking, are also risk factors for CRC [1,2,3]. CRC is categorized into four distinct stages. These stages provide information about the extent/spread of the disease and guide treatment decisions [5,6,7]:

- Stage 0: This is the earliest stage of CRC. At this stage, the abnormal cells are confined to the mucosa of the colon or rectum and have not spread to nearby tissues.
- Stage I: The primary polyps have grown through the mucosa of the colon or rectum and may have invaded the muscle layer. However, they have not spread to the lymph nodes or distant sites.
- Stage II: The cancer has spread beyond the muscle layer and may have invaded nearby tissues. However, it has not reached the lymph nodes or distant organs. Stage II CRC tumors are further classified into stages II a, b, and c, regarding the cancer spread to the serosa or nearby organs.
- Stage III: The cancer has spread to nearby lymph nodes but has not metastasized to distant sites. Stage III CRC tumors are further subdivided into stages III a, b, and c, regarding the cancer spread to the serosa, inner and middle layers of the colon, and neighboring lymph nodes.
- Stage IV: This is the most advanced stage of CRC. At this stage, the cancer has metastasized to distant organs, such as the liver, lungs, or other parts of the body.

The diagnosis of colon cancer involves a combination of screening tests, diagnostic imaging, and pathological analysis. Common screening methods include colonoscopy, flexible sigmoidoscopy, digital rectal exam, and stool-based tests, such as fecal immunochemical test (FIT) and fecal occult blood tests (FOBTs), which look for genetic material (i.e., blood or DNA) in the stool. Colonoscopy is currently the most widely used and cost-effective technique for detecting CRC and taking biopsies for further histopathological evaluation [1,3,6]. Artificial intelligence techniques have been integrated into colonoscopy procedures to increase their effectiveness in detecting and evaluating colorectal polyps [3,8,9]. The implementation of a computer-aided diagnostic (CAD) system using deep-learning technology has shown promising results in accurately determining polyp histology (from the range of 63.8–71.8% to the range 82.7–84.2%) [3,8]. In addition, the use of narrow-band imaging (NBI) in colonoscopy can also improve polyps detection relative to white light colonoscopy (accuracy 95% vs. 74%) [3]. When conventional colonoscopy cannot be performed or is contraindicated or rejected by patients, colon capsule endoscopy (CCE) offers an alternative option for screening patients at moderate risk of CRC. CCE is an ingestible, wireless, and disposable capsule that takes multiple pictures of the colon, allowing for a painless and radiation-free study of the entire colon without sedation or gas insufflation. Although CCE has shown promise as a screening tool, it is important to note that it is not as comprehensive as colonoscopy in terms of detecting smaller polyps or providing therapeutic interventions. CCE is not recommended as a first-line screening or diagnostic method for CRC [3,9,10]. Diagnostic imaging techniques such as computed tomography (CT) and magnetic resonance imaging (MRI) are used to evaluate the extent of tumor involvement and detect metastasis [11]. Tissue samples obtained through biopsy or surgical resection are examined histologically to confirm the presence of cancer and determine its stage [3].

The treatment of CRC depends on various factors, including the stage of the disease, location of the tumor, and the overall health of the patient. The conventional treatment modalities include surgery, chemotherapy, radiotherapy, and immunotherapy [2,6,12]. Table 1 shows the treatment modality commonly used at each stage of CRC [1,5,6,7]. Surgical resection (open or laparoscopic) of the tumor is the main curative treatment option. Overall, these therapies are typically most effective when the disease is diagnosed at an early stage (approximately 90% survival rate). However, when CRC is detected at a late stage, patients often experience a poor prognosis (approximately 15% survival rate in stage 4), indicating the need for improved detection methods and more effective treatment options [2,6].

Despite the availability of several conventional treatment approaches for CRC, these methods often have limitations, such as severe side effects, invasiveness, and limited efficacy in late-stage CRC [6]. Photodynamic therapy (PDT) is an emerging minimally invasive treatment that shows promise in improving CRC treatment outcomes. PDT involves the administration of a photosensitizing agent, which selectively accumulates in the cancer cells. Subsequent exposure to light at specific wavelength activates the photosensitizer (PS), leading to the production of reactive oxygen species (ROS) that cause localized cell death and tumor destruction [13,14]. In the case of colon cancer, PDT offers several advantages. First, it is a minimally invasive procedure that can be performed during endoscopic procedures, such as colonoscopy, allowing for targeted treatment directly at the tumor site. This localized approach reduces the potential damage to healthy tissues around the tumor. Another advantage is the ability to administer multiple dosages with minimal side effects. Unlike some conventional treatments, which can cause serious side effects due to their systemic nature, PDT can be repeated without cumulative toxicity. This flexibility in dosage administration allows for more effective treatment plans [6].

## 2. Principles of PDT and Photosensitizers

**Figure 1:**
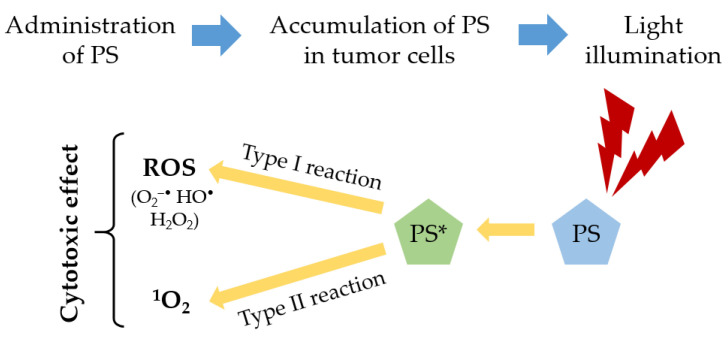
Principles of PDT.

PDT is a non-invasive modality that can be used to treat various types of cancers effectively. The therapy involves the integration of three key components: PS, light at specific wavelength, and oxygen [13,14,15]. First, a PS is administered to the patient (topically or intravenous), which selectively accumulates in the tumor tissues. After a period of time, called “drug-light interval”, the tumor region is illuminated by a specific light source, typically in the red spectral region (λ ≥ 600 nm), and the PS becomes activated. This light source is carefully chosen to match the absorption properties of the PS, allowing for optimal activation. Upon exposure to the light, the activated PS undergoes a photochemical reaction with the molecular oxygen surrounding the tumor [13,16,17]. This reaction generates cytotoxic singlet oxygen (^1^O_2_) and other ROS, such as superoxide radical (O_2_^−•^), hydroxyl radical (HO^•^), and hydrogen peroxide (H_2_O_2_), which are highly destructive to the tumor tissues. These ROS cause oxidative damage to the tumor cells, leading to their destruction and subsequent tumor regression [13,17,18]. Two types of photodynamic reaction can occur in PDT. The type I reaction occurs when the excited state of the PS (PS*) reacts directly with a substrate, such as a cell membrane or a molecule, leading to hydrogen atom abstraction or electron transfer reactions. This interaction results in the generation of free radicals and radical ions. These radicals can react with other molecules, e.g., molecular oxygen, producing ROS [13,14,17,18,19]. The type II reaction occurs when the excited state of the PS transfer energy directly to the molecular oxygen, forming the singlet oxygen. Approximately all PSs have a high quantum yield in this reaction [13,14,18,19]. The relative contributions of type I and type II reactions to PDT can vary depending on several factors, such as the PS properties, oxygen concentration, and the binding affinity of PS to the substrate. Understanding and optimizing both types of photodynamic reactions are important for maximizing the therapeutic outcomes of PDT [13,14,19,20]. Figure 1 illustrates the principles involved in PDT.

The products resulting from both photodynamic reactions lead to tumor destruction and the overall therapeutic effect of PDT via three interrelated mechanisms: direct cytotoxic effects on tumor cells, indirect damage to the tumor-associated vasculature, and induction of an inflammatory response and activation of an immune response [14,18,20]. The reactive species generated during the photodynamic reactions can directly damage the tumor cells. These reactive species can induce cellular stress, disrupt cellular components, and trigger apoptotic pathways, leading to programmed cell death (apoptosis) or cell death by other mechanisms (necrosis) [13,15,17,20]. Photodynamic reactions can also affect tumor-associated vasculature. The reactive species, particularly singlet oxygen, can damage the blood vessels supplying the tumor, leading to vascular rupture and the subsequent deprivation of oxygen and nutrients to the tumor cells. This indirect damage to the tumor-associated vasculature contributes to the overall destruction of the tumor [13,17,20]. An inflammatory response in the treated area can also be induced. The cellular damage caused by PDT triggers the release of inflammatory mediators and the recruitment of immune cells. This inflammatory response can further enhance the destruction of tumor cells and contribute to the activation of the immune system against the tumor. The immune response can recognize and target the tumor cells, leading to immune-mediated clearance and potentially providing long-term protection against tumor recurrence [13,20,21].

Oxygen plays a crucial role in the production of ROS during PDT. Tumor tissues often have an altered microenvironment with reduced levels of oxygen (hypoxia), affecting the effectiveness of PDT. Innovative strategies have been developed to overcome hypoxia-related limitations and improve the effectiveness of PDT. These strategies aim to increase oxygen levels in the target tissue, either by improving local oxygen generation (e.g., H_2_O_2_-decomposition, water-splitting, and photosynthetic oxygen production) or by increasing the oxygen-carrying capacity of the blood (e.g., perfluorocarbons and hemoglobin). Fractionated PDT can also help in tumor hypoxia, i.e., delivering light in multiple fractions instead of all at once [13,14,18,21].

The choice of light source for PDT depends on the specific location of the cancerous tissue and the PS used. Commonly used light sources include lasers and lamps; however, there is a growing trend towards the use of laser-emitting diodes (LEDs). Interestingly, even natural sunlight has been used as a light source in a variation of PDT known as daylight PDT [13,14,22,23]. Table 2 shows the main advantages and disadvantages of the light sources used in PDT. Light can penetrate biological tissues with minimal absorption and scattering at the tissue optical window (600–1200 nm), allowing for deeper tissue penetration. However, wavelengths greater than approximately 850 nm generally contain insufficient energy to generate a strong photodynamic effect and require solutions such as the upconversion of photons for sufficient singlet oxygen quantum yields. Thus, the phototherapeutic window predominantly used in PDT ranges from 600 to 850 nm [13,14,22]. In addition to this window, there are two other significant biological windows in the near-infrared (NIR) spectrum, known as NIR-II (1000–1350 nm) and NIR-III (1500–1800 nm). These additional windows also offer advantages as they demonstrate reduced auto-fluorescence, light scattering, and light absorption. NIR light proves to be more advantageous compared to visible light when dealing with tissue depths greater than 0.5 mm [24].

PSs are substances that are capable of absorbing light at specific wavelengths and triggering photochemical reactions [14]. An ideal PS should demonstrate high purity and chemical stability, selective tumor targeting, low dark toxicity, strong absorption with a high molar extinction coefficient (ε) for higher light wavelengths (600 to 800 nm), high singlet oxygen quantum yield (Φ_Δ_), and rapid clearance from the body [14,17,29]. PSs can be categorized into three generations based on their complexity and successful application outcomes [6,13,14]. First-generation PSs includes hematoporphyrin derivative (HpD) and porfimer sodium. These early PSs were derived from porphyrins and exhibited broad absorption spectra but had limited selectivity, low molar extinction coefficient, and prolonged skin photosensitivity [6,18]. Second-generation PSs aimed to improve upon the limitations of first-generation compounds. These PSs were often synthetic modifications of porphyrin and chlorin structures, leading to enhanced selectivity, increased phototoxicity, and reduced skin photosensitivity. Examples of second-generation PSs include chlorins, protoporphyrin IX (PpIX), benzoporphyrins, hypericin, phthalocyanines, and 5-aminolevulinic acid (5-ALA) [6,16]. Third-generation PSs have emerged with improved tumor selectivity. This is achieved through the incorporation of targeting molecules (antibody conjugation) or encapsulation into carriers (such as nanoparticles or liposomes), enhancing their specificity for tumor regions. These advancements allow for more precise and targeted photodynamic therapy, maximizing the therapeutic effect while minimizing off-target effects [6,13,14]. Table 3 and Table 4 show the PSs approved for clinical applications in PDT and some PSs under clinical investigation, respectively.

Today, PS progresses towards the improvement of PDT specificity and efficacy, involving the use of porous carriers for sensitizers, such as liposomes [33], silica nanoparticles [34,35], polymers [36,37,38], metallic nanoparticles [39,40,41], quantum dots [42,43,44], and carbon nanomaterials [45,46], that can be encapsulated into a large number of PS [15,22,47,48]. Table 5 shows the main properties of the nanoparticles used as carriers of PSs in PDT.

## 3. PDT and CRC

The increased resistance of tumor cells to conventional chemotherapeutic and biologic drugs used in CRC treatment, along with their non-specific toxicity to healthy tissues, highlights the need for alternative therapeutic approaches. One such approach is PDT, which offers several advantages in the treatment of CRC [51]:

- Minimally invasive treatment [15,52];
- Minimization of damage to healthy tissues, reducing the risk of systemic side effects, through the targeted and localized approach of PDT [6,51,52];
- Overcoming the issue of multidrug resistance encountered with conventional chemotherapy, as PSs preferentially accumulate in CRC cells [51,52];
- Activation of immune responses against CRC. The release of tumor-associated antigens and the induction of immunogenic cell death triggered by PDT can stimulate an antitumor immune response, leading to the destruction of residual tumor cells and providing long-term therapeutic benefits [53].

In recent years, extensive preclinical and clinical research has been conducted on PDT for the treatment of CRC. This research has yielded valuable insights into the potential of PDT as a therapeutic approach for CRC. Moreover, there has been growing interest in combining PDT with other treatment modalities, such as surgery and radiotherapy. By integrating PDT with established treatment methods, a synergistic effect can be achieved, leading to improved outcomes for patients with CRC [32].

### 3.1. Preclinical Research

#### 3.1.1. In Vitro Studies

Most preclinical studies investigating the potential application of PDT in colon and rectal cancer have focused on assessing the phototoxic effects of PSs on in vitro cultured colorectal tumor cells. One notable advantage of in vitro methods is the ability to directly use human cells, eliminating the need for translation from animal to human. These in vitro studies serve as a valuable starting point for evaluating the efficacy and selectivity of different PSs in targeting and destroying colorectal tumor cells. By exposing tumor cells to PSs and subsequent light activation, researchers can assess the cytotoxic effects and determine the optimal conditions for PDT treatment. In these preclinical studies, various parameters are investigated, including the choice of PS, optimal concentration, light dose, and treatment duration. Additionally, these in vitro studies provide insights into the underlying mechanisms of PDT in CRC. Researchers investigate the cellular and molecular responses triggered by PDT, such as apoptosis, necrosis, and the generation of ROS. Understanding these mechanisms is crucial for optimizing PDT protocols and developing more effective treatments [51,54]. Monolayer cultures, while valuable for investigating treatment effects, lack the complexity needed to replicate the heterogeneous nature of in vivo conditions. To address these limitations, three-dimensional tumor models have emerged as a partial solution, allowing for long-term studies of single-model tumors and single cells overtime. Three-dimensional tumor models provide a more realistic representation of the tumor microenvironment, incorporating factors such as cell–cell interactions, extracellular matrix components, and nutrient gradients. However, it is important to recognize that three-dimensional tumor models also have their limitations. They do not fully replicate the complexity of in vivo tumor growth, metastasis, and immunological interactions. Additional factors, such as a lack of vasculature or immune cell infiltration, may affect the translation of findings to clinical settings [54]. Table 6 shows some preclinical in vitro studies of PDT performed in colorectal tumor cells.

#### 3.1.2. Animal Studies

In vitro studies are essential for establishing the foundations of PDT in CRC research; however, further investigations are needed to validate these findings in animal models and eventually in clinical trials. A prerequisite for starting a clinical trial is evidence of a positive impact of the technique or drug on animals. The complexity of in vivo tumor microenvironments and the potential influence of factors such as blood flow, immune response, and tissue architecture require further studies to assess the full potential of PDT in the treatment of colon and rectal cancer [51,85]. The choice of animal model is therefore very important and should mimic the human situation as much as possible. The most commonly studied animals are rats and mice [85,86]. The selection of an appropriate cell line is another crucial aspect. Many pharmacological studies use nude animals bearing human-derived tumors [85]. Nude animals, lacking a functional immune system, are commonly used to avoid immune rejection of human tumor cells. Due to the greater susceptibility of these mice to infections, the cell lines must be free of mouse pathogens and the mice must be maintained under specific pathogen-free conditions [86,87]. Human tumor cells are cultured in vitro and then injected directly into the animal, usually subcutaneously, on the desired tumor location. A large number of human CRC cell lines grown as xenograft tumors at a subcutaneous location in nude mice have been subjected to PDT [86]. The advantages of this model include relatively rapid tumor development and easy observation. It allows the evaluation of genes and signaling pathways that drive tumor growth. The most important drawbacks, however, are the lack of immune response, no infiltration of adjacent tissues, and rarely observed metastases. To overcome some of those obstacles, the cells are sometimes implanted orthotopically [88,89,90]. Table 7 shows some preclinical animal studies of PDT performed in colorectal tumors.

Spontaneous tumors, called autochthonous, can be generated via the administration of carcinogens (chemicals; viruses; or physical stimuli, e.g., UV radiation) [85,86]. These models effectively recapitulate the time-dependent and multistage progression of tumor formation in response to relevant environmental carcinogens and tumor-promoting agents [86,89]. However, they are extremely time-consuming, with a very low reproducibility rate, and can also pose exposure risks to personnel handling the animals [85].

The chorioallantoic membrane (CAM) assay using fertilized chicken eggs is a straightforward and intermediate experimental model situated between in vitro cell culture and laboratory in vivo animal studies [86,87]. This method involves removing a small window in the shell of a fertilized chicken egg to access the underlying chorioallantoic membrane [86]. This model allows the growth of tumor cells that are applied as a suspension on the surface of the membrane, transforming into tumors that develop their own blood supply through the process of angiogenesis [86,87]. PS can be injected into the blood vessels or topically applied to the xenografted tumors on the CAM [86]. This model has the advantage of simplicity of operation, cost-effectiveness, and ethical issues being relatively simplified compared to other in vivo models. However, it remains sparse and poorly characterized compared to murine models [85,86,87].

Animal experiments in the field of PDT research serve multiple purposes, including clarifying mechanisms underlying the observed photodynamic effects at the organism level, assessing PDT safety and efficacy, and translating these findings into potential clinical benefits [51].

### 3.2. Clinical Trials

Clinical trials have been crucial to ensuring the safe and effective development of medical interventions since the Medical Research Council trial in 1948 which demonstrated the effectiveness of streptomycin in the treatment of tuberculosis [51]. Clinical PDT treatment involves the application of visible light that is combined with a PS and oxygen to destroy CRC cells in patients [6,12]. Due to the lack of standardized guidelines for the use of PDT in patients with CRC, clinical trials have employed a wide range of PDT parameters: choice of PS and its concentration, type and dose of light, PDT application regimen, and compatibility with conventional therapeutic methods [51]. Typically, the clinical trials of PDT in the treatment of CRC are performed with optical fibers from the endoscope to deliver the necessary light for PS excitation. Thus, PDT selectively damages colon cancerous tissues, minimizing undesirable side effects and systemic cytotoxicity to adjacent healthy cells [12,108]. Table 8 shows some clinical trials of PDT in CRC. Most studies exploring the application of PDT in CRC are pilot, phase I, and phase II clinical trials. Phase I trials involve small groups of patients in advanced stages of the disease and are primarily focused on assessing the safety and toxicity of PDT. Phase II trials involve larger groups of patients and aim to investigate the clinical efficacy of PDT [51]. There are limited data available from phase III clinical trials evaluating the overall efficacy of PDT in CRC. Currently, there are no phase IV clinical trials conducted for PDT in CRC, as it is not a registered method for this specific type of cancer. However, the conducted clinical trials have consistently demonstrated the effectiveness of PDT in clinical application for CRC treatment [12,51].

## 4. Challenges and Limitations of PDT in CRC

Despite the many positive features of PDT in the treatment of CRC, the clinical application of this treatment has encountered certain challenges, particularly regarding PS water solubility, selective tumor uptake, and the difficulty of treating deep tumors due to low tissue penetration of the illuminating light [12,50,108]. Another limitation is its effectiveness only in the treatment of non-hypoxic tumors. The cytotoxic mechanism of action of PDT depends on the presence of oxygen, making it less suitable for hypoxic tumor environments [5,13,14].

The presence of cancer stem cells, which have a high resistance to PDT, has been associated with the recurrence and progression of CRC [12,73]. Therefore, additional therapeutic strategies may be required to target advanced types of CRC, including both primary tumors and secondary systemic disease [12].

The hydrophobicity of PS poses a challenge in PDT, as insoluble PSs tend to aggregate during administration. This aggregation hampers effective cellular uptake into target malignant tissues and reduces the production of high levels of ROS, limiting the overall efficiency of PDT [5,108]. To achieve maximum levels of ROS generation and ensure complete tumor destruction in PDT, it is crucial to successfully deliver and localize high concentrations of PS drugs in target tumor tissues [52,125]. However, in clinical settings using first- and second-generation PS drugs, poor outcomes and effectiveness have been observed. This is because only small amounts of PS drugs are able to overcome biological barriers in the human body and passively accumulate in tumor cells, resulting in low levels of ROS generation and limited tumor destruction [52,108]. Moreover, this passive accumulation can sometimes lead to the accumulation of PS drugs in healthy tissues, causing unwanted side effects, such as photosensitivity and damage to normal tissues [108,125].

To overcome the challenges mentioned above, third-generation PSs with nanoparticle carriers (such as liposomes, dendrimers, polymeric nanoparticles, and inorganic nanoparticles) are currently being investigated to increase the water solubility and cellular uptake of PS, ensuring more efficient and targeted delivery to the tumor site and overall efficacy of PDT in CRC [5,12,52,108]. The use of nanoparticles-based PS carriers has great potential to advance the field of CRC treatment and improve patient outcomes [12].

NIR light has better penetration efficiency for deep tissue compared to visible light. However, longer wavelengths contain insufficient energy to generate a strong photodynamic effect. To overcome this limitation, several studies have suggested using two-photon NIR photodynamic activation and upconversion-mediated photodynamic activation [24,126,127]. In two-photon NIR photodynamic activation, the PS is excited by the simultaneous absorption of two lower-energy photons within the NIR spectrum, where the sum of the photon energies equals the bandgap energy of the PS, thus allowing deeper penetration of light and less photo-bleaching of PS in tissues [24,127]. An alternative method involves the use of upconversion nanoparticles (UCNPs) to mediate NIR photodynamic activation. These nanoparticles have the ability to absorb multiple photons at a specific wavelength and subsequently convert them into a single photon via an anti-Stokes shift. This converted photon has a shorter wavelength, resulting in higher energy content, which can be effectively employed to excite a PS in the PDT [22,24]. In the last few years, several UCNPs have been created. Gao et al. developed UCNPs loaded with ZnPc as a PS and conjugated to c(RGDyK) for the targeting of the tumor vasculature and achieved a deep-tissue PS activation by NIR light irradiation [128]. In other studies, Ce6-loaded UCNPs [129,130], MC540-loaded UCNPs [129], and AgBiS2-loaded UCNPs [131] have been synthesized, inducing significant tumor growth inhibition after PDT at high wavelengths for upconversion [22].

Light delivery to the CRC can sometimes be difficult. Rodrigues et al. proposed the innovative integration of a PDT module into the endoscope capsule to minimally invasively deliver light to the CRC and perform PDT [29].

## 5. Combined Therapies: Synergistic Approaches to Enhance PDT Efficacy in CRC

Extensive evidence suggests that CRC exhibits complex heterogeneity within specific mutations, thus posing challenges for many existing treatment approaches [12,132]. Conventional monotherapies commonly used in the treatment of CRC have shown limited success in completely eradicating colorectal cells and are often accompanied by unwanted side effects. Consequently, there is growing interest in exploring combination therapies that offer synergistic effects and overcome the limitations of single treatments [5,12]. The use of combined therapies holds great promise, as they offer improved efficacy and reduced side effects compared to monotherapies. This approach seeks to capitalize on the benefits of combining multiple treatment modalities to effectively target CRC and enhance treatment outcomes [5,12].

PDT has shown the ability to induce immunogenic cell death, a form of cell death that activates immune responses and promotes antitumor immunity. This property of PDT makes it a potential candidate for therapies combined with immunotherapies that enhance the host’s immune system. One such immunotherapy approach is the use of immune checkpoint inhibitors. These are antibodies that block the suppressive immune checkpoint mechanisms, allowing the immune system to respond more strongly against cancer cells. By combining PDT with immune checkpoint inhibitors, the goal is to increase the immune response and improve the overall therapeutic outcome [12,133]. Recently, a large number of nanoparticles have been explored as promising delivery vehicles for PDT combined with immune checkpoint inhibitors for tumors to enhance PDT treatment efficiency. He et al. conjugated nanoscale coordination polymer (NCP) core–shell nanoparticles that carried oxaliplatin in the core and the PS pyrolipid in the shell (NCP@pyrolipid). The integration of oxaliplatin chemotherapy, PDT, and checkpoint blocking therapy enhanced antitumor immunity and exhibited effective therapeutic effects for the treatment of metastatic CRC [134]. Xu et al. simultaneously loaded UCNPs with Ce6 and imiquimod (R837), a Toll-like-receptor-7 agonist. The obtained multitasking UCNP-Ce6-R837 nanoparticles under NIR irradiation showed effective photodynamic destruction and promoted strong antitumor immune responses in CT26 cells [135]. Yuan et al. used multifunctional nanoparticles loaded with photosensitized mTHPC (mTHPC@VeC/T-RGD NPs)-mediated PDT treatment to potentiate the antitumor efficacy of PD-L1 blockade for CRC treatment and investigate the underlying mechanisms of PDT enhancing PD-L1 blockade therapeutic effect in this combination therapy [133].

Chemotherapy coupled with surgery can significantly increase the survival of patients with metastatic CRC. However, chemotherapy often comes with a range of side effects that can greatly impact the quality of life of patients with CRC [136]. The combination of PDT and chemotherapy has been investigated as a potential treatment approach for CRC. When combined, PDT and chemotherapy may offer several advantages. First, PDT can be used to selectively target and destroy cancer cells in a localized manner, reducing the need for extensive chemotherapy, which affects healthy tissues. This targeted approach may help minimize the side effects associated with systemic chemotherapy. Second, the cytotoxic effects of PDT can enhance the effectiveness of chemotherapy by sensitizing cancer cells to the action of chemotherapy drugs. This synergistic effect may improve tumor response rates and potentially overcome drug resistance. Su et al. purposed a chemo-photodynamic therapy nanoplatform capable of manipulating redox homeostasis and boosting endoplasmic reticulum stress against CRC by integrating the chemotherapeutic agent brigatinib with PS Ce6 into a TPGS-based nanosystem [137]. Hashemkhani et al. proposed the use of Cetuximab-conjugated Ag_2_S quantum dots loaded with ALA/5 fluorouracil to achieve tumor-specific targeting for PDT/chemotherapy combination therapy in EGFR(+) CRC cell lines [138]. Chen et al. proposed a mixture of porphyrin-grafted lipid/camptothecin–floxuridine triad microbubbles converted via ultrasound as a combined therapeutic strategy for CRC. The aim was to combine chemotherapy promoted by camptothecin–floxuridine with PDT promoted by the porphyrin-grafted lipid to overcome CRC multidrug resistance [139].

The combination of photothermal therapy (PTT) and PDT has shown promise in the treatment of CRC due to the cytotoxic ROS and hyperthermia that are generated by PSs under light exposure [12]. Seo et al. synthesized methylene blue-loaded gold nanorod@SiO_2_ nanoparticles for synergistic therapy of CRC combining PDT and PTT [140]. Wang et al. designed hyaluronic-acid-decorated polydopamine nanoparticles with conjugated Ce6 for PDT/PTT cancer-targeting therapy. The synergetic effects of the compound demonstrated increased accumulation within tumors, increased tumor growth inhibition, and improved phototoxic effect in HCT-116 tumor-bearing mice [141]. Yang et al. produced sub-100 SN-38-encapsulated photonic micelles for effective trimodal (photothermal-, photodynamic-, and chemotherapy) cancer therapy, demonstrating dramatically increased in vivo antitumor efficacy over single treatment in nude mice bearing an HT-29 colon cancer xenograft [142].

Table 9 summarizes the advantages and disadvantages of therapies used in synergistic approaches to enhance PDT efficacy in the treatment of CRC.

## 6. Conclusions

The overall prognosis of CRC tends to be very poor due to the challenges associated with its diagnosis using conventional methods. The lack of sensitivity in these approaches often leads to diagnoses only during advanced stages of the disease. The effectiveness of conventional treatments for CRC is highly dependent on the stage, size, and progression of the tumor. Early detection of premalignant colorectal tumors is the best chance of increasing patient survival rates. Despite efforts to develop these conventional treatment methods to combat CRC, they often result in adverse effects that can affect overall treatment outcomes.

PDT stands out as a remarkably safe alternative when compared to surgical, chemotherapy, and radiotherapy procedures. Its exceptional ability to selectively accumulate PSs in tumor cells ensures that the cytotoxic impact is only limited to pathological cells. With its high selectivity and action focused on a small area, PDT ensures predictable depth, making it an undeniable attribute of this method. Unlike other existing oncology therapies, PDT offers a unique combination of safety, low invasiveness, and repeatable application, without the significant risk of complications such as intestinal-wall perforation or mutagenic reactions. However, like any treatment modality, PDT has its limitations: low depth of light penetration into tissues and effective PSs biodistribution in CRC tumors. To overcome these limitations, third-generation PSs with nanoparticle carriers are currently being investigated. This approach aims to enhance the water solubility and cellular uptake of PS, enabling more efficient and targeted delivery to the tumor site and ultimately improving the overall effectiveness of PDT in CRC. The use of nanoparticles-based PS carriers holds significant potential to advance the field of CRC treatment and improve patient outcomes.

There are many positive and promising research studies being conducted in preclinical and clinical trials for the use of PDT in CRC treatment. Although PDT is not currently employed as a clinical treatment for early forms of CRC, it undeniably represents a significant ray of hope for a substantial group of patients seeking minimally invasive palliative interventions. PDT not only has the potential to prolong life but also to improve the overall comfort of these individuals. Several clinical studies have shown promising results for the use of PDT in CRC. These results show a glimpse of potential beyond preclinical studies, showing its efficacy in less advanced tumors and in the palliative treatment of advanced lesions. However, more research is needed to optimize treatment protocols, determine the ideal PSs and light parameters, and evaluate the long-term efficacy and safety.

Ongoing research in PDT includes the development of more refined PSs, improvement of light delivery systems, and development of combined therapies to enhance the effectiveness of PDT. By harnessing the potential of PDT, researchers and clinicians strive to improve patient survival rates, minimize treatment side effects, and ultimately provide better therapeutic options for individuals with colon cancer. This research will allow the development of specific guidelines for the use of PDT in CRC.

In summary, PDT has the potential to emerge as a rival competitor to conventional therapies in the field of CRC treatment. The future integration of PDT into routine CRC treatments in clinical practice is foreseen, either as part of a multimodal approach or as a single treatment against early cancer or palliative care.

## Figures and Tables

**Table 1:** Treatment of CRC at different stages.

Stage of CRC	Treatment
Stage 0	Surgical removal of the polyp or abnormal tissue through endoscopic procedures or minimally invasive surgery
Stage I	Surgical resection of the tumor
Stage II	Surgical resection of the tumor. Adjuvant chemotherapy depending on specific tumor characteristics and overall health of the patient
Stage III	Surgical resection of the tumor and lymph nodes, followed by adjuvant chemotherapy
Stage IV	Monotherapy or a combination of chemotherapy, biologic targeted therapy, immunotherapy, palliative surgery, radiotherapy, and radiofrequency ablation

**Table 2:** Advantages and disadvantages of the light sources used in PDT [13,14,16,23,25,26,27,28].

Light Source	Advantages	Disadvantages
Laser	High light intensity Monochromatic light Efficient coupling to optical fibers	Expensive Bulky
Lamps	Low cost Portable Easy to use Wide illumination field	Thermal effect Wide spectral width Needs optical filtering Low-light intensity Limited to easily accessible places
LEDs	Low cost Small Thermally nondestructive Available in flexible arrays	Less powerful (compared to laser) Large beam divergence Broad spectral width
Daylight	Cheaper Minimal patient discomfort Shorter clinical visits	Scheduling difficulty Difficult to control light exposure

**Table 3:** PSs clinically used in PDT [13,14,15,16,22,30,31].

Photosensitizer	Wavelength (nm)	Approval	Applications
Porfimer sodium (Photofrin^®^)	630	Worldwide	Esophageal cancer, Barrett’s esophagus, and non-small cell lung cancer
5-aminolevulinic acid (Levulan^®^/Ameluz^®^)	635	Worldwide	Actinic keratosis and superficial basal cell carcinoma
Methyl aminolevulinate (Metvix^®^/Metvixia^®^)	570–670	Worldwide	Actinic keratosis and basal cell carcinoma
Verteporfin (Visudyne^®^)	690	Worldwide	Age-related macular degeneration
Temoporfin (Foscan^®^)	652	Europe	Advanced head and neck cancer
LUZ11 (Redaporfin^®^)	749	Europe	Biliary tract cancer
Padeliporfin (TOOKAD^®^)	753	Europe	Prostate cancer
Hexyl-aminolevulinate (Hexvix^®^/Cysview^®^)	360–450	Europe, USA, Canada	Bladder cancer detection
Talaporfin sodium (Laserphyrin^®^)	664	Japan	Lung and esophageal cancers and brain tumors

**Table 4:** PSs under clinical investigation [13,14,30,31,32].

Photosensitizer	Wavelength (nm)	Applications
Radachlorin^®^	662	Skin cancer
Photochlor^®^	664	Head and neck cancer
Purlytin^®^	664	Age-related macular degeneration
Fotolon^®^	665	Nasopharyngeal sarcoma
Photosens^®^	670	Lung, liver, breast, skin, and gastrointestinal cancer
Lutrin^®^	732	Coronary artery disease

**Table 5:** Main properties of the nanoparticles used as carriers of PSs in PDT [15,47,49,50].

Nanoparticle	Properties
Liposomes	Delivery of hydrophobic agents Good biocompatibility and biodegradability
Silica nanoparticles	High biocompatibility and biodegradability Highly hydrophilic Easy surface functionalization Trigger ROS production
Polymers	Biocompatibility Delivery of hydrophobic agents High permeability through cell membranes Loading of multiple agents
Metallic nanoparticles	Amplification of PS excitation Enhance ROS production Surface modification to bind to PS
Quantum dots	Photostability Light-re-emitting properties High quantum yields
Carbon nanoparticles	High immobilization of PSs Water solubility Biocompatibility

**Table 6:** Preclinical in vitro studies of PDT in colorectal tumor cells.

Ref.	Tumor Cell Line	Photosensitizer	Irradiation Conditions	Year
[55]	HT-29	Porfimer sodium, 2.5–10 μg/mL	585 nm, 9.2 W/m^2^, 2700 J/m^2^	2001
[56]	Colo 201	Temoporfin, 0.125–1 μg/mL	500 nm, 7 mW/cm^2^, 1–15 J/cm^2^	2002
[57]	HCT-116	Phthalocyanine Pc 4, 0–300 nM	670–675 nm, 200 mJ/cm^2^	2005
[58]	HT-29	Hypericin, 0.04–0.1 μM	530–620 nm, 4.4 J/cm^2^	2006
[59]	HCT-116	PpIX ^1^, 0.5–10 μg/ml	633 nm, 2 J/cm^2^	2007
[60]	LoVo	Pyropheophorbide-a or verteporfin conjugates with scFvs ^2^, 0.25–100 μM	680 nm, 13.4 J/cm^2^	2008
[61]	HT-29	SN-38-loaded CSBC ^3^ micelles, 0.001–1000 μg/mL	660 nm, 19.5 mW/cm^2^, 7 J/cm^2^	2009
[62]	HCT-116	Newly synthesized phenyl porphyrin derivatives, temoporfin, 1 μg/mL	White light, 20 mW/cm^2^ 630 nm, 0.6 mW/cm^2^	2009
[63]	SW-480	TCPP ^4^, TCPP nanoparticles or TCPP-loaded PLGA ^5^ nanoparticles, 1 μM	400–440 nm, 141 mW/cm^2^, 15 J/cm^2^	2009
[64]	HT-29	Ce6-aptamers ^6^, 0.1–100,000 nM	664 nm, 20–30 mW/cm^2^, 12 J/cm^2^	2009
[65]	HCT-116	DH-II-24, 5 μg/mL	630 nm, 1.45 mW/cm^2^, 0.02–0.17 J/cm^2^	2009
[66]	LoVo	Porfimer sodium, 15–30 μg/mL	633 nm, 3–6 J/cm^2^	2010
[67]	HT-29	Pheophorbide a, 0–2 μM	630 nm, 2 J/cm^2^	2010
[68]	HCT-116	PpIX silica nanoparticles, 5 μM	630 nm, 4 mW/cm^2^	2010
[69]	HT-29, HCT-116	H_2_TFPC-SGlc or Talaporfin sodium, 1 μM	633 nm, 37 mW/cm^2^, 16 J/cm^2^	2011
[70]	CaCo-2	GaPcCl ^7^, 2–100 μg/mL	661 nm, ≈90 mW/cm^2^, 2.5–8.5 J/cm^2^	2012
[71]	DLD-1	ZnPcS_mix_ ^8^, 5–40 μM	680 nm, 5 J/cm^2^	2012
[72]	WiDr	TPPS_2a_ ^9^, 0.1 μg/mL	435 nm, 13.5 mW/cm^2^	2013
[73]	HT-29	PpIX, 1 μg/mL	633 nm, 1 and 5 J/cm^2^	2014
[74]	C-26	Ce6, 0.5 μg/mL	662 nm, 105 mW/cm^2^, 3–12 J/cm^2^	2015
[75]	SW-620	5-ALA, 3 mM	630 nm, 60 mW/cm^2^, 4.5 J/cm^2^	2016
[76]	SW-620, SW-480	Temoporfin, 0.18–11.76 μM	650 nm, 60 mW/cm^2^, 1.5–6 J/cm^2^	2017
[77]	HCT-116	PMMA@PorVa ^10^, 0.1–100 nM	Visible light, 158.4 J/cm^2^	2018
[29]	RKO, HCT-15	Temoporfin, 0.5–10 μg/mL	653 nm, 11 mW/cm^2^, 2.5–10 J/cm^2^	2019
[78]	HT-29	PGL NPs ^11^, 0–8 μM	650 nm, 200 mW/cm^2^	2020
[79]	CT-26	Ce6, 0.1–1.8 μM PI3Kγ ^12^ inhibitor IPI-549, 0.5–9.3 μM	660 nm, 800 mW/cm^2^, 48 J/cm^2^	2021
[80]	HCT-116	BC4 ^13^, 0–100 μM	761 nm, 30 mW/cm^2^, 48 J/cm^2^	2022
[81]	CaCo-2	ZnPcS_4_/Ag@mSiO_2_, 0–0.5 μM	674 nm, 9.5 mW/cm^2^, 10 J/cm^2^	2022
[82]	CaCo-2	AlClPcTS41, 0.125–0.75 μM	636 nm, 10 J/cm^2^	2023
[83]	HCT-15	Porphyrin-based photosensitizers (0–50 μM) + low dose of doxorubicin (0.5 μM)	600–720 nm, 50 mW/cm^2^, 20 J/cm^2^	2023
[84]	HCT-116	CFN-gel ^14^, 0–5 μM	660 nm, 50 mW/cm^2^, 9 J/cm^2^	2023

^1^ Protoporphyrin IX; ^2^ single-chain variable fragment; ^3^ chlorin-core star-shaped block copolymer; ^4^ meso-tetra (carboxyphenyl) porphyrin; ^5^ poly (lactic-co-glycolic acid); ^6^ chlorin e6; ^7^ gallium (III) phthalocyanine chloride; ^8^ zinc sulfophthalocyanine; ^9^ meso-tetraphenylporphine with two sulphonate groups on adjacent phenyl rings; ^10^ poly-methyl methacrylate nanoparticles covalently loaded with the porphyrin; ^11^ porphyrin-grafted lipid nanoparticles; ^12^ phosphoinositide 3-kinase gamma inhibitor IPI-549; ^13^ meso-tetrakis [1-(2′-bromoethyl)-3-pyridyl]-bacteriochlorin tetrabromide; ^14^ fucoidan-based theranostic nanogel.

**Table 7:** Preclinical animal studies of PDT in colorectal tumors.

Ref.	Animal Model	Photosensitizer	Irradiation Conditions	Year
[91]	Female nu/nu–athymic mice xenografted with SW-480 tumor cells	Phthalocyanine Pc4, 1 mg/kg intravenously (i.v.)	670 nm, 150 mW/cm^2^, 150 J/cm^2^	2000
[92]	Male athymic nude mice bearing HT-29 tumor cells	Porfimer sodium or liposomal pheophorbide a, 30 mg/kg intraperitoneally (i.p.)	636 or 665 nm, 200 mW/cm^2^ or 150 mW/cm^2^, 100 J/cm^2^	2002
[93]	Female C57BL/6NCr mice bearing Colo-38 tumor cells	NPe6 ^1^, 5 mg/kg i.v.	664 nm, 9 mW/cm^2^, 61 J/cm^2^	2005
[94]	Female BALB/c and C57Bl/6 mice xenografted with CT-26 tumor cells	ATX-S10 Na(II), 5 mg/Kg i.v.	670 nm, 150 J/cm^2^	2006
[95]	Female BALB/c–nu/nu athymic nude mice bearing WiDr tumor cells	PP(Arg)_2_^2^, 2 and 10 mg/kg i.v.	632 nm, 250 mW/cm^2^, 150 J/cm^2^	2007
[65]	Female BALB/c nude mice bearing HCT-116 tumor cells	DH-II-24, 1 mg/kg i.v.	> 630 nm, 154 J/cm^2^	2009
[96]	Female BALB/c, BALB/nude and NOD/scid mice bearing CT-26 tumor cells	WST11, 9 mg/kg i.v.	755 nm, 100 mW/cm^2^, 30 J/cm^2^	2009
[69]	Female BALB/c Slc-nu/nu nude mice xenografted with HT-29 and HCT-116 tumor cells	H_2_TFPC-SGlc or Talaporfin, 6.25 μmol/kg i.v.	633 nm, 37.5 J/cm^2^	2011
[97]	Female BALB/c mice bearing CT-26 tumor cells	Hypericin, 50 or 200 μg i.v.	600 nm, 27 or 50 mW/cm^2^, 14 or 60 J/cm^2^	2011
[98]	Female Swiss nude/nude mice xenografted with HT-29 tumor cells	5,10,15-tri{para-O-[2-(2-O-α-_D_-Manosyloxy)-ethoxy]-ethoxy-phenyl}-20-phenyl porphyrin, 0.6 mg/kg i.v.	650 nm, 75 J/cm^2^	2012
[99]	Female BALB/c nude mice bearing HT-29 tumor cells	5-ALA, 250 mg/kg i.p.	456 nm or white light or 635 nm, 96 mW/cm^2^, 32 J/cm^2^	2013
[100]	BALB/c nude mice xenografted with HCT-116 tumor cells	PPA-stent membranes ^3^ (40 μg/cm^2^ Pheo-A)	670 nm, 100 J/cm^2^	2014
[74]	Male BALB/cByJ mice bearing C-26 tumor cells	Ce6, 2.25 mg/kg i.v.	662 nm, 95 mW/cm^2^, 100 J/cm^2^	2015
[101]	Female BALB/c and BALB/c Slc-nu/nu mice xenografted with CT-26 tumor cells	G-chlorin, 1.25 μmol/kg i.v.	660 nm, 49 mW/cm^2^, 40 J/cm^2^	2016
[102]	HT-29 tumor-bearing mice	Ce6 or HANP/Ce6 ^4^, 5 mg/kg i.v.	630 nm, 150 mW/cm^2^, 270 J/cm^2^	2017
[103]	BALB/c nude mice bearing CT-26 tumor cells	Ce6-PVA ^5^, 5 mg/kg i.v.	658 nm, 100 mW/cm^2^, 150 J/cm^2^	2018
[104]	Female BALB/c-nu/nu athymic nude mice bearing HT-29 tumor cells	Temoporfin, 0.3 mg/kg i.v. Bevacizumab, 5 mg/kg i.p.	652 nm, 100 mW/cm^2^, 10 J/cm^2^	2018
[105]	Female BALB/c nude mice bearing HT-29 tumor cells	TPPOH ^6^, 3.26 mg/kg i.v. TPPOH-X SNPs ^7^, 1.16 mg/kg of TPPOH and 334 mg/kg of SNPs i.v.	660 nm, 200 J/cm^2^	2019
[78]	Male BALB/c nude mice bearing HT-29 tumor cells	200 μL of PGL NPs i.v. (2 mg/mL)	650 nm, 200 mW/cm^2^, 120 J/cm^2^	2020
[106]	Male BALB/c nude mice bearing CT-26 tumor cells	Pc9-T1107 ^8^, 35 μg/kg i.v.	650 nm, 306 mW/cm^2^, 500 J/cm^2^	2020
[79]	Male BALB/c nude mice bearing CT-26 tumor cells	Ce6, 0.75 mg/kg i.v. PI3Kγ inhibitor IPI-549, 3 mg/kg i.v.	660 nm, 800 mW/cm^2^, 48 J/cm^2^	2021
[107]	Female C57BL/6 J mice bearing MC38 tumor cells	ZnPc-EVs ^9^, 400 μM in 100 μL PBS i.v.	690 nm, 333 mW/cm^2^, 100 J/cm^2^	2022
[84]	Male BALB/c nude mice bearing HCT-116 tumor cells	5-ALA, 250 mg/kg i.v. Ce6, 5 mg/kg i.v. CFN-gel, 5 mg/kg i.v.	660 nm, 50 mW/cm^2^, 18 J/cm^2^	2023

^1^ N-aspartyl chlorin e6; ^2^ di-L-arginine protoporphyrinate; ^3^ pullulan acetate-conjugated pheophorbide A; ^4^ encapsulation of chlorin e6 into a hyaluronic acid nanoparticle; ^5^ chlorin e6 conjugated to polyvinyl alcohol; ^6^ 5-(4-hydroxyphenyl)-10,15,20-triphenylporphyrin; ^7^ silica nanoparticles coated with xylan-TPPOH conjugate; ^8^ lipophilic phthalocyanine encapsulated into T1107 poloxamine micelle; ^9^ extracellular vesicles containing zinc phthalocyanine.

**Table 8:** Clinical trials of PDT in CRC.

Ref.	Phase	Case (Patient Number)	Photosensitizer	Irradiation Conditions	Year
[109]	I/II	Palliative advanced rectal cancer (6)	Porfimer sodium, 2 mg/kg i.v.	630 nm, 50–200 J/cm^2^	1991
[110]	Pilot	Colorectal adenomas (8)	HpD, 2.5 mg/kg i.v. Porfimer sodium, 2 mg/kg i.v.	630 nm, 100 mW, 50 J	1994
[111]	Pilot	Duodenal and colorectal polyps (6)	5-ALA, 30–60 mg/kg orally Porfimer sodium, 2 mg/kg i.v.	628 nm, 50 or 100 J	1995
[112]	I	Different malignant tumors (11)	Npe6, 0.5–3.5 mg/kg i.v.	664 nm, 25—100 J/cm^2^	1998
[113]	Pilot	Tumors in esophagus, duodenum and rectum (22)	m-THPC, 0.15 mg/kg i.v. Porfimer sodium, 2 mg/kg i.v. 5-ALA, 60 mg/kg orally	650 nm, 10—15 J/cm^2^ (m-THPC) 628 nm, 50—150 J/cm^2^ (porfimer sodium and 5-ALA)	1998
[114]	Pilot	Different malignant tumors (51)	Radachlorin, 0.8–1.2 mg/kg	662 nm, 100–500 J/cm^2^	2002
[115]	Pilot	Rectal cancer (2)	HpD, 2.5 mg/kg i.v.	627.8 nm, 150—280 mW	2003
[116]	Pilot	Anal intraepithelial neoplasia (1)	20% 5-ALA cream, topically	630 nm, 125 mW/cm^2^, 125 J/cm^2^	2003
[117]	I	Different malignant tumors (21)	Talaporfin sodium, 40 mg/m^2^ i.v.	660 nm, 250—2000 J, 50 mW	2003
[118]	I	Liver metastases from colorectal carcinoma (8)	m-THPBC ^1^, 3 or 6 mg/kg i.v. Talaporfin sodium, 40 mg/m^2^ i.v.	740 nm, 60 J/cm (m-THPBC) 664 nm, 100 J/cm (talaporfin)	2004
[119]	I	Liver metastases from colorectal carcinoma (24)	m-THPBC, 0.3—0.6 mg/kg i.v.	740 nm, 60 J/cm	2005
[120]	II	Peritoneal carcinomatosis and sarcomatosis (100)	Porfimer sodium, 2.5 mg/kg i.v.	532 nm, 150 mW/cm^2^, 2.5 J/cm^2^	2006
[121]	II/III	Anal cancer (8)	Porfimer sodium, 1.2 mg/kg i.v.	630 nm, 300 J/cm + 200 J/cm^2^	2010
[122]	Pilot	Anal intra-epithelial neoplasia (15)	5-ALA cream, topically Porfimer sodium, 1.2 mg/kg i.v.	630 nm, 75 J/cm^2^ (in two cycles topically) or 100 J/cm^2^ (systemically)	2014
[123]	II/III	Advanced CRC (23)	Porfimer sodium, 2 mg/kg i.v.	630 nm, 200 J/cm^2^	2016
[124]	Pilot	Rectal adenocarcinoma (1)	Porphyrin, 2 mg/kg i.v.	630 nm, 100 mW/cm^2^, 120 J/cm^2^	2019

^1^ 5,10,15,20-tetrakis(m-hydroxyphenyl) bacteriochlorin.

**Table 9:** Advantages and disadvantages of therapies used in PDT synergistic approaches in the treatment of CRC.

Therapy	Advantages	Disadvantages
PDT	Spatiotemporal selectivity Immunogenicity Limited or no drug resistance	Limited light penetration Oxygen dependence
Immunotherapy	Light independence Memory effect Immune-cell harnessing	Low response rate Immune-related side effects
Chemotherapy	Light independence Many available drugs Most clinically used anticancer therapy	Systemic toxicity Multidrug resistance
PTT	Spatiotemporal selectivity Immunogenicity Oxygen independence	Limited light penetration Heat-shock response

## Data Availability

Not applicable.
